# Use of whole body vibration therapy in individuals with moderate severity of cerebral palsy- a feasibility study

**DOI:** 10.1186/s12883-019-1307-5

**Published:** 2019-05-01

**Authors:** Tamis W. Pin, Penelope B. Butler, Sheila Purves

**Affiliations:** 1 Department of Rehabilitation Sciences, The Hong Kong Polytechnic University, Hung Hom, Hong Kong; 2 Manchester Metropolitan University, Manchester, UK

**Keywords:** Cerebral palsy, Whole body vibration, Functional abilities, Static standing, Balance

## Abstract

**Background:**

This pilot study was to examine the feasibility and tolerance of whole body vibration therapy (WBVT) for children and adults with moderate severity of cerebral palsy (CP) being graded as levels III or IV on the Gross Motor Function Classification Scale (GMFCS).

**Methods:**

Study participants received the additional WBVT when standing still on the vibration platform for three 3-min bouts of vibration (20 Hz, 2 mm amplitude), 4 days per week for 4 weeks. In addition to questions relating to feasibility and participants’ opinions, assessment at baseline and completion of the intervention included the Gross Motor Function Measure-66 Item Set (GMFM-66 IS), 2-min walk test (2MWT), Timed Up and Go test (TUG) and Pediatric Evaluation of Disability Inventory (PEDI). Wilcoxon Signed Ranks test was used to compare the results.

**Results:**

Fourteen participants (mean age = 25.25 years SD 3.71; 9 males, 64%; GMFCS level III *n* = 13, 92%) were recruited and completed the study. The attendance rate was over 90% with no adverse events. All participants tolerated the protocol which was satisfactorily delivered in a clinical setting.

**Conclusions:**

The present WBVT protocol was feasible, safe and well-tolerated by the participants with moderate severity of CP, justifying future studies with larger samples and more rigorous study design.

**Trial registration:**

The present study has been registered under the ClinicalTrials.gov (NCT03375736) and the date of registration commenced on 18 December 2017.

## Background

Cerebral palsy (CP) is one of the most common childhood motor disabilities [1]. Although the brain damage is non-progressive, the clinical features resulting from the upper motor neuron lesion cause spasticity, poor motor control, muscle weakness and poor balance. All these features lead to secondary musculoskeletal changes resulting in limb joint contractures and bony deformities which further impact muscle strength and motor function [2]. When compared with their typically developing healthy peers, children and young adults with CP are thus not able to perform the same amount of exercise to improve their muscle strength and motor function [3].

Individuals with CP are commonly classified into one of five levels according to their physical mobility using the Gross Motor Function Classification System (GMFCS) levels I to V^1^ [4]. Individuals of GMFCS levels III or IV, whose balance and functional mobility are greatly compromised, have the greatest limitation in performing exercises to improve their muscle strength and motor function [1].

Mechanical vibration has been used as an intervention strategy for neuro-modulation in various neurological diseases [5, 6]. The vibrations stimulate the alpha-motor neurons of the muscle spindles leading to the tonic vibration reflex [6, 7]. The reflex then increases voluntary muscle contractions [8]. It has also been shown that the vibrations stimulate peripheral mechanoreceptors, possibly inducing neuroplasticity via somatosensory and motor pathways if applied repeatedly [9]. Furthermore, vibration that is provided to the whole body may increase neural drive to the muscles, allowing recruitment of previously inactive motor units [8] with resulting increase in muscle mass and strength.

The vibration can be provided to the whole body or as a focal vibration to a specific muscle or tendon [6]. Previous studies have shown the effectiveness of vibration in reducing spasticity in patients after stroke or spinal cord injuries, in stimulating proprioception and subsequently better motor control during walking in patients with stroke and Parkinson disease, and in enhancing muscle activity during walking in patients with spinal cord injuries [6]. Preliminary findings have shown that vibration may possibly improve the gait pattern for individuals with childhood ataxia [10]. In whole body vibration therapy (WBVT), the user stands in a static position or performs some dynamic movements on a device providing vibrations from a few Hz to 50 Hz (Hz, Hertz represents the number of complete up and down movement cycle per second) [8].

Research on WBVT for both children and adults with CP have appeared to show positive trends in terms of gross motor function, balance, muscle strength and muscle tone [5, 11–14]. There is some suggestion that WBVT improved muscle strength, reduced spasticity, and improved gait parameters and functional activities for children and adolescents with CP [9–13] with no report of any adverse event. However, most studies have included only participants with mild severity of CP. In addition, almost all studies required the study participants to perform simple exercises on the vibration platform [5, 11]. As far as can be determined, only two published studies have been conducted on children and young adults with more severe CP, i.e. GMFCS level V and these focused only on the bone mineral density of the participants [15, 16]. Thus, the level of evidence of benefit of WBVT for individuals with moderate severity of CP (GMFCS levels III and IV) remains unknown, as does the practicality of conducting WBVT for these individuals.

This pilot study aimed to examine the feasibility of a 4-week WBVT programme on children and adults with moderate severity of CP. The specific feasibility objectives were:

i. To investigate the practicality and tolerance of the intervention protocol adapted from the study by Gusso and colleagues [17]. Their study showed positive results on muscle strength and walking ability in young adults with CP of GMFCS levels II and III after 20 weeks but it is noted that this was an uncontrolled study [17].
ii. To investigate if any change in functional abilities and balance resulted from static standing on the vibration platform.
iii. To investigate if there were any adverse effects of this intervention for this population group.
iv. To gain information about the study participants’ feelings regarding this intervention in terms of comfort and personal opinion.

## Methods

A convenience sample of a total of 10 individuals aged between 6 and 45 years was targeted, recognizing that sample size calculation is not usually required for pilot studies [18]. This broad age range allowed examination of the feasibility of WBVT on a wide spectrum of this population group. The inclusion criteria were participants: (i) with a diagnosis of CP; (ii) with moderate severity of CP, i.e. GMFCS levels III or IV; (iii) able to stand for 3 min independently or with own hand support on rails; (iv) able to understand simple instructions; and (v) able to tolerate clinical examination. The exclusion criteria were participants: (i) with bone fracture 8 weeks prior to enrolment to the present study, or with acute thrombosis, muscle or tendon inflammation, renal stones, discopathy or arthritis as reported by the participants and/or their parent/guardian; (ii) with metal implants in their spine or lower limbs; (iii) using anabolic agents or growth hormone for at least 1 month, within 3 months prior to enrolment into the present study; and (iv) being pregnant.

The study participants received the WBVT when standing still on the Galileo Med L Plus model 2000 (Novotech Medical GmbH, Germany), wearing their normal clothing and footwear. WBVT was additional to their usual therapy program. The WBVT intervention sessions consisted of three 3-min bouts of vibration of 20 Hz and a peak-to-peak amplitude of 2 mm with a 3-min rest in between based on the study by Gusso and colleagues [17]. The parameters of the vibration were gradually progressed to the desired level in the first seven sessions allowing the study participants to accustom to the intense sensory stimulation from the vibration (Table 2). The intervention took place on 4 days per week for 4 weeks.

All the participants were assessed at baseline and completion of the intervention using the following outcome measures:

i. Gross Motor Function Measure (GMFM-66) Item Set (IS) [19] is a validated and reliable assessment of general gross motor function in various positions including lying, sitting, 4-point kneeling, high kneeling and standing for individuals with CP [20]. The GMFM-66 IS is a condensed version of the GMFM to improve its efficiency of administration in clinical settings [19].
ii. Two-minute walk test (2MWT) to assess submaximal exercise capacity. The distance covered in 2 min was measured using a distance-measuring trundle wheel following the published testing protocol [21, 22];
iii. Timed Up and Go (TUG) test to assess balance and functional mobility [23, 24];
iv. A validated Chinese version of the Pediatric Evaluation of Disability Inventory (PEDI) [25, 26] to assess functional capacities in the domains of daily activities (73 items), mobility (59 items) and social/cognitive function (65 items); and
v. Visual analogue scale to assess any discomfort associated with the intervention. The scale ranged from 0 (no discomfort) to 10 (extreme discomfort), as reported by the study participants and/or by their carers’ proxy. This was accompanied by any subjective comments the participants wished to make.

Ethics approval was granted from the affiliated University of the first and third authors. An informed consent form was signed by the participants over 18 years old, or if not, including all participants under 18 years old, by their parents/guardians. This study was registered under the ClinicalTrials.gov (NCT03375736).

The study results were presented as means and standard deviations. Due to the small sample size, non-parametric Wilcoxon Signed Ranks test was used to compare the results before and after the WBVT. The statistical significance level was set at *p* < 0.05.

## Results

**Fig. 1:**
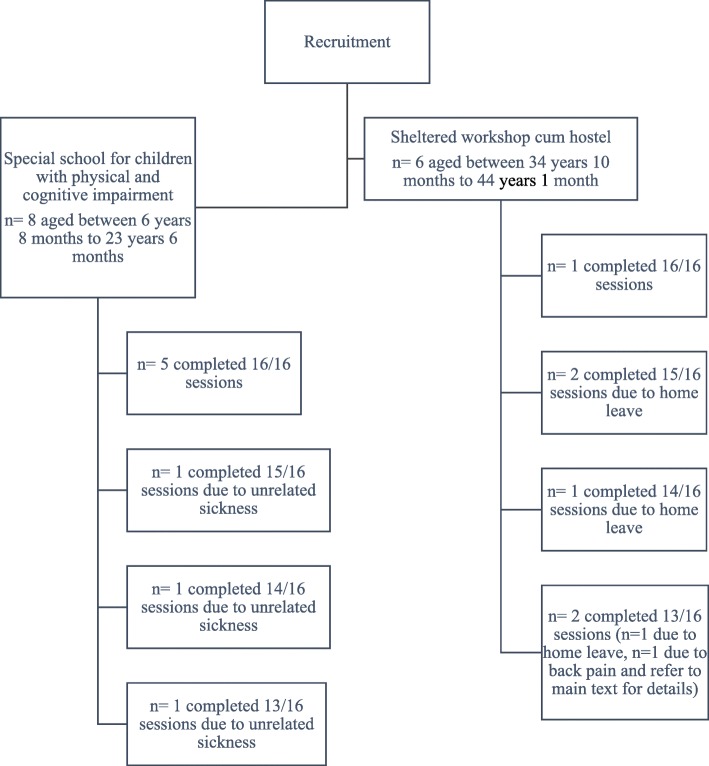
Participants flowchart

Fourteen participants (mean age = 25.25 years SD 3.71; 9 males, 64%) were recruited from a special school for students with physical and cognitive impairment (*n* = 8, mean age = 14.56 years, SD 6.78; 5 males, 63%) and a sheltered workshop cum hostel (*n* = 6, mean age = 39.50 years, SD 3.31; 4 males, 67%) (Fig. 1). An overwhelming response from the participants and the on-site therapists led to a larger sample size than originally planned. Table 1 shows the characteristics of the study participants. The majority of participants functioned at the GMFCS level III (*n* = 13, 92%).

**Table 1:** Characteristics of study participants

	Age group (yr)	Dx	GMFCS	GMFM-66 IS		2MWT (meters)		TUG (seconds)		PEDI					
										Self-care		Mobility		Social function	
				Pre	Post	Pre	Post	Pre	Post	Pre	Post	Pre	Post	Pre	Post
VT01	41–45	Ataxia	III	50.6	50.9	44	39.6	63	80	47	47	28	28	54	54
VT02	31–35	Sp dip	III	49.9	49.9	74.7	61.2	34	40	56	57	32	32	57	57
VT03	41–45	Sp dys quad	III	50.9	51.6	73.1	75.2	42	52	59	61	52	52	65	65
VT04	35–40	Sp dys quad	II/III*	55.4	55.9	93.1	109.5	19	19	61	61	32	32	30	30
VT05	35–40	Sp L hemi	III	50.1	50.1	37.8	40.1	42	41	61	65	39	40	62	62
VT06	35–40	Sp dys quad	II/III*	52.6	55.2	71.5	86.7	10	10	58	58	56	56	60	60
VT07	6–10	Ataxia	II/III*	62.7	65.6	58.3	63.1	18	18	39	40	38	38	39	39
VT08	6–10	Sp dys quad	III	55.9	58.1	69.5	72.7	20	17	58	58	37	37	59	59
VT09	6–10	Sp dip	III	51.3	52.9	55.3	64.7	34	27	61	61	42	42	60	60
VT10	11–15	Equi to sp. quad	III	44.6	42.4	9.9	9.6	192	136	36	36	17	18	37	37
VT11	16–20	Sp quad	IV	39	39	NA	NA	NA	NA	42	42	6	10	61	61
VT12	16–20	Sp tri	III	59.1	56.2	33.7	37.5	32	27	66	68	45	47	60	60
VT13	16–20	Sp dip	III	53.1	52.6	60.5	65.1	21	21	69	69	36	36	48	48
VT14	21–25	Equi sp. quad	III	47.3	50.3	54.1	38.2	25	29	42	42	36	36	31	31

*2MWT* 2-min walk test, *Dip* Diplegia, *dys* Dystonia, *Dx* Diagnosis, *equi* Equivalent to, *GMFM-66 IS* Gross Motor Function Measure 66 Item Set, *hemi* Hemiplegia, *L* Left, *PEDI* Pediatric Evaluation of Disability Inventory, *quad* Quadriplegia, *Sp* Spastic, *TUG* Timed up and go test, *tri* Triplegia

*These 3 participants had mixed clinical features of GMFCS levels II and III. They all relied on use of a walking aid with supervision (VT7) or wheeled mobility (VT4 and VT6) for long distances in outdoor environments. All were able to walk without a walking aid for short distances in indoor environments but their balance raised safety concerns; hence, VT7 used a walking frame most of the time. Neither VT4 nor VT6 were able to hold any walking aid due to the dystonic features in their upper limbs

NA- data not available as VT11 used a body-weight-support walking frame for therapeutic walking only and required moderate manual assistance

Two employed physical therapists separately provided the WBVT to all the study participants at their respective recruitment sites. The attendance rate of the study participants was 93.36% (SD 7.61). The greatest number of missed intervention sessions was three out of 16 sessions for three participants (VT2, VT4 and VT14 in Table 1 and Fig. 1). Six participants completed all 16 sessions. One adult participant (VT4) stumbled when he stepped off the vibration platform after the eighth session. This participant missed the following two sessions of WBVT and requested to re-start the WBVT in the third week. No adverse event was reported for any other participant. All participants coped well with the protocol and the physical therapists had no difficulty in delivering the program.

**Fig. 2:**
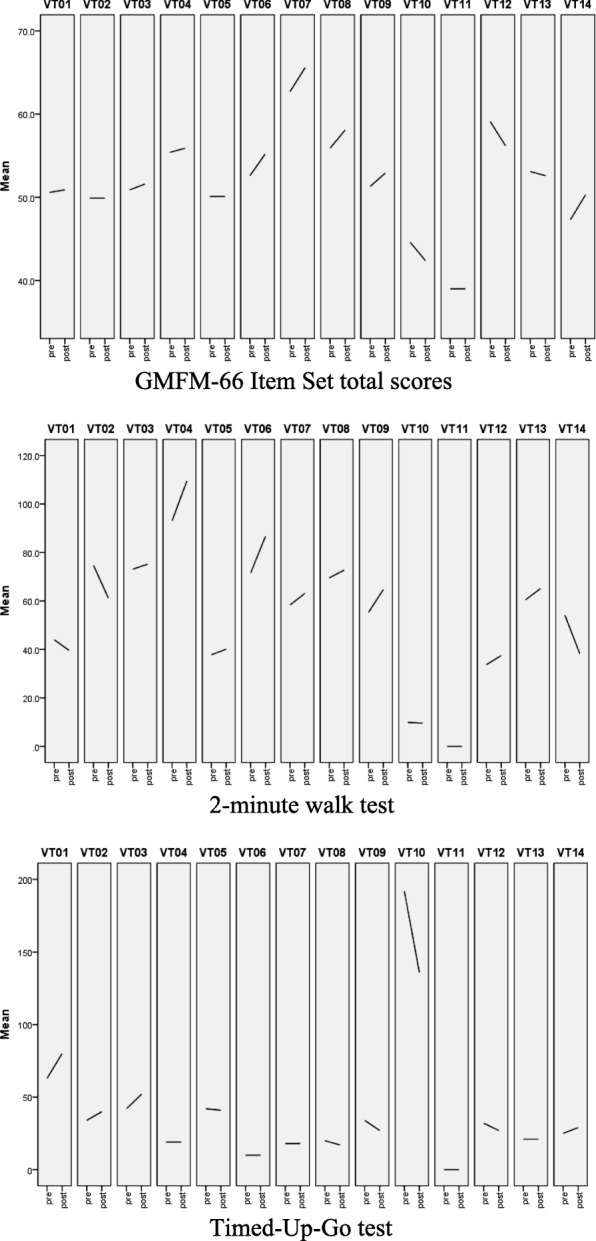
Graphed study results

All the participants were assessed by the first author (TWP) at baseline and after 4 weeks of intervention. The results are presented in Table 1 and graphically in Fig. 2. The Wilcoxon Signed Rank test showed no significant difference in any of the outcome measures.

### Availability of data and materials

The raw data and data sets used and analyzed in the present study are available from the corresponding author on reasonable request.

## Discussion

This pilot study examined the feasibility of an adapted WBVT protocol for children and adults with moderate severity of CP. The study also investigated any changes in functional abilities and balance.

### Feasibility of the intervention protocol

Most of the previous studies on WBVT required the participants to perform simple exercises, such as mini-squats or lunges, on the vibration platform. This poses a great challenge for individuals with moderate severity of CP but these are the individuals most vulnerable to poor muscle strength and motor function as a consequence of their greatly compromised mobility [27, 28]. The present study thus investigated the feasibility of static standing on the vibration platform with both knees in slight flexion and with hand support if needed. This protocol (Table 2) was less demanding than the one recommended for children and adolescents with disabilities [5]. Participants tolerated the intervention well and had high completion percentage with the minimal number of reported adverse events: this suggests that the present protocol of the WBVT was feasible, safe and acceptable for the study participants across the given age range. Physical therapists were able to deliver the WBVT without difficulty in a routine clinical environment.

### Functional abilities and balance

This feasibility study used the same outcome measures on study participants across a wide age range. Both the 2MWT and TUG have been conducted on both children and adults with the test instructions largely remaining the same [21, 23, 24]. Although the GMFM-66 has been validated only for children with CP, it has also been used as an outcome measure for adults with CP [29–32]. The GMFM-66 IS is a condensed version of the GMFM-66. The PEDI is an outcome measure designed to assess functional performance in daily activities for children aged 9 months to 7.5 years of age but can be used above this age if typical performance has not been achieved [26] as in previous research studies for older individuals [33–35].

In general, small but positive changes were shown in gross motor function (GMFM-66 IS), functional mobility (2MWT), self-care activities (PEDI self-care), and mobility activities (PEDI mobility) (Table 1 and Fig. 2). The improvement in the PEDI self-care activities reached statistical significance and, in mobility activities, a close to statistical significance. This small pilot study is insufficient to verify whether static standing on the WBVT platform confers the same benefit as exercises during WBVT but may indicate a preliminary trend. It was interesting to note that the self-care activities that showed improvement all involved core stability and balance in various positions, such as cleaning or drying the body after a shower, putting toothpaste on the toothbrush and brushing the teeth, or spilling water. Previous studies have shown that muscle strength around the trunk and lower limbs was increased with WBVT, which in turn, improved core stability and reduced dependence on hand support [12, 14, 17, 29]. Participants also found moving in and out of positions easier (PEDI mobility). Participant VT11 showed the largest increase in this score (Table 1 and Fig. 2). He was a young man with spastic quadriplegic CP and the only participant with GMFCS level IV. He stated that he could roll, get in and out of a chair and bed with more ease and with no increase in carer assistance.

### Adverse effects

No participant complained of pain or discomfort during or after the WBVT. We cannot confirm that the stumble experienced by Participant VT4 was linked to the WBVT as he had a history of unexplained falls.

### Subjective comments from study participants

The most frequent comments by the study participants after the WBVT included ‘very relaxed’ legs (7 out of 14 participants, 50%) and ‘jelly’ legs or ‘weak’ legs (5 out of 14 participants, 36%). Some vocal adult participants (VT2 and VT12) indicated that their legs felt less stiff. Two participants (VT3 and VT6), both residents in the sheltered workshop cum hostel, commented on the change in walking pattern of Participant VT4 after one of the intervention sessions. VT4 usually walked using a total extensor pattern but used a flat-foot gait pattern after that session. Inclusion of 3D gait analysis, where practical, could be valuable in future studies or video-recording with analytical tools if kinematic and kinetic measures are unavailable.

### Limitations of present study

This pilot study was with a small sample size and a low level of study design. Repetition of the outcome measures at 4 weeks was a relatively short duration that could have led to some learning effect. However, the primary focus of this study was to ascertain the feasibility of the WBVT program.

The study group were mostly of GMFCS level III and only one participant of GMFCS level IV. Potential participants with more severe CP were cognitively unable to follow instructions to remain still in the required posture on the vibration platform. This difficulty may be overcome in future studies by providing WBVT on a standing tilt-table with an installed vibration platform.

Overall, the present preliminary findings justify future studies with more rigorous study designs and larger samples that include more participants with greater severity of CP (GMFCS level IV) before the present WBVT protocol is recommended for use in clinical settings.

## Conclusions

The present pilot study has shown that the proposed WBVT treatment protocol is feasible, safe and acceptable for children, adolescents and adults with moderate severity of CP and is practical in a routine clinical setting. There were small trends towards improvement in functional abilities and balance with this static standing protocol. These findings justify use of the same protocol on a larger sample size of this population group using a more rigorous study design.

## Appendix

**Table 2:** Intervention protocol of the present study

Session	Vibration 1	Rest 1	Vibration 2	Rest 2	Vibration 3	Rest 3
1st	1 min; 12 Hz, 1 mm	3 min	1 min; 12 Hz, 1 mm	3 min	1 min; 15 Hz, 1 mm	3 min
2nd	1 min; 15 Hz, 1 mm	3 min	1 min; 15 Hz, 1 mm	3 min	2 min; 15 Hz, 1 mm	3 min
3th	2 min; 15 Hz, 1 mm	3 min	3 min; 15 Hz, 1 mm	3 min	3 min; 15 Hz, 1 mm	3 min
4th	2 min; 18 Hz, 1 mm	3 min	2 min; 18 Hz, 1 mm	3 min	2 min; 18 Hz, 1 mm	3 min
5th	3 min; 20 Hz, 1 mm	3 min	3 min; 20 Hz, 1 mm	3 min	3 min; 20 Hz, 1 mm	3 min
6th	3 min; 20 Hz, 1 mm	3 min	3 min; 20 Hz, 2 mm	3 min	3 min; 20 Hz, 2 mm	3 min
>7th	3 min; 20 Hz, 2 mm	3 min	3 min; 20 Hz, 2 mm	3 min	3 min; 20 Hz, 2 mm	3 min

Adapted from the study by Gusso and colleagues (2016)

## Abbreviations

2MWT: 2-min walk test
CP: Cerebral palsy
GMFCS: Gross Motor Function Classification Scale
GMFM-66 IS: Gross Motor Function Measure 66 Item Set
PEDI: Pediatric Evaluation of Disability Inventory
TUG: Timed-up-go test
WBVT: Whole body vibration therapy

## Footnotes

1 In general, individuals of levels I and II ambulate unaided and are able to perform more advanced motor skills such as running and jumping with limitations. Individuals of level III mobilise with hand-held mobility devices such as crutches or walking frames. Individuals of levels IV have very limited functional mobility and are mostly limited to an indoor environment. Individuals with level V are non-ambulatory or totally rely on wheeled mobility aids in all environment settings.
